# Transcriptome analysis reveals candidate genes related to phosphorus starvation tolerance in sorghum

**DOI:** 10.1186/s12870-019-1914-8

**Published:** 2019-07-11

**Authors:** Jinglong Zhang, Fangfang Jiang, Yixin Shen, Qiuwen Zhan, Binqiang Bai, Wei Chen, Yingjun Chi

**Affiliations:** 1 College of Agro-grassland Science, Nanjing Agricultural University, Nanjing, 210095 Jiangsu Province China; 2 College of Agriculture, Anhui Science and Technology University, Fengyang, China

**Keywords:** Sorghum, Transcriptome analysis, Phosphorus starvation, Candidate genes, Malate, Root development

## Abstract

**Background:**

Phosphorus (P) deficiency in soil is a worldwide issue and a major constraint on the production of sorghum, which is an important staple food, forage and energy crop. The depletion of P reserves and the increasing price of P fertilizer make fertilizer application impractical, especially in developing countries. Therefore, identifying sorghum accessions with low-P tolerance and understanding the underlying molecular basis for this tolerance will facilitate the breeding of P-efficient plants, thereby resolving the P crisis in sorghum farming. However, knowledge in these areas is very limited.

**Results:**

The 29 sorghum accessions used in this study demonstrated great variability in their tolerance to low-P stress. The internal P content in the shoot was correlated with P tolerance. A low-P-tolerant accession and a low-P-sensitive accession were chosen for RNA-seq analysis to identify potential underlying molecular mechanisms. A total of 2089 candidate genes related to P starvation tolerance were revealed and found to be enriched in 11 pathways. Gene Ontology (GO) enrichment analyses showed that the candidate genes were associated with oxidoreductase activity. In addition, further study showed that malate affected the length of the primary root and the number of tips in sorghum suffering from low-P stress.

**Conclusions:**

Our results show that acquisition of P from soil contributes to low-P tolerance in different sorghum accessions; however, the underlying molecular mechanism is complicated. Plant hormone (including auxin, ethylene, jasmonic acid, salicylic acid and abscisic acid) signal transduction related genes and many transcriptional factors were found to be involved in low-P tolerance in sorghum. The identified accessions will be useful for breeding new sorghum varieties with enhanced P starvation tolerance.

**Electronic supplementary material:**

The online version of this article (10.1186/s12870-019-1914-8) contains supplementary material, which is available to authorized users.

## Background

Phosphorus (P) is an essential macronutrient for plant growth and development and is considered to regulate energy metabolism, enzymatic reactions, and signal transduction processes [1, 2]. Inorganic orthophosphate (Pi) is the primary source of P for plants. However, Pi can be readily fixed with aluminum and iron in acidic soil and with calcium in alkaline soil [3]. Since Pi content in soil is too low to satisfy the requirements for plant growth, excessive quantities of P fertilizer must be applied, which possibly cause environmental pollution [4, 5]. The global rock phosphate reserve is a nonrenewable resource, and approximately 70% of cultivated lands worldwide suffer from P deficiency [6]. Therefore, the development of plants that are better adapted to low-P environments is a sustainable and economical approach in agricultural production.

To adapt to persistent P deficiency, plants have developed several strategies, including changing root morphology [7], exuding organic acids and phosphatases [6], and establishing symbiotic relationships with arbuscular mycorrhizal fungi [8]. These strategies in plants are dependent on changes in gene expression. Numerous genes related to low-P stress have been identified and recently reported in plants [9–11]. The *phosphate transporter1* (*PHT1*) gene family participates in the uptake of Pi from the soil. As a member of the PHT1 family, *PHT1;1* plays an important role in Pi uptake from the soil [12–15]. Moreover, many transcription factors (TFs) could regulate the expression of *PHT1;1*, such as phosphate starvation response 1 (PHR1) [16], WRKY75 [17], WRKY45 [18] and MYB domain protein 62 (MYB62) [19]. The PHO1 family plays an important role in Pi translocation from root to shoot [20–22] and is downregulated by WRKY6 and WRKY42 [23, 24]. Additionally, miR399, miR827 [25–27] and the zinc finger TF ZAT6 [28] modulate phosphate homeostasis. Most of these factors were identified in model plants, such as Arabidopsis and rice. In sorghum, however, only the multiple homologs of the *phosphorus starvation tolerance 1* (*PSTOL1*) gene have been identified, which may be associated with changes in root morphology and root system architecture under low-P conditions [29].

Rice, maize, wheat and sorghum are important graminaceous crops. Among them, P uptake by sorghum was only lower than that by rice [30]. Furthermore, sorghum is not only a staple food for more than 500 million people but also a popular forage crop [29, 31, 32]. Given its high yield, extensive use and excellent adaptation to harsh environmental conditions, sorghum was planted specifically in arid and semiarid regions [33, 34]. It has been suggested that the grain yield of sorghum is highly correlated with P levels [35], and approximately 0.2% of the dry weight of this crop is contributed by P [36]. Although many genes and signal pathways related to low-P stress have been identified in rice [37], wheat [38] and maize [39] through transcript profiling, the genes and pathways related to low-P stress in sorghum remain unclear.

In this study, the P starvation tolerance of 29 sorghum accessions was evaluated. Accessions 12484 and 13443 were identified as low-P-tolerant and low-P-sensitive accessions, respectively. The gene expression in the roots of the two accessions, which both underwent P treatment for 8 days, was analyzed by mRNA sequencing (RNA-seq). Differentially expressed genes (DEGs) related to P starvation tolerance were identified through transcriptome profiles. Gene ontology (GO) and Kyoto Encyclopedia of Genes and Genomes (KEGG) analyses were performed. Moreover, the effect of malate application on root development in sorghum was investigated. The findings reported in this work increase our understanding of the molecular mechanisms of P starvation in sorghum.

## Methods

### Pot experiments

To evaluate the P starvation tolerance of the 29 sorghum accessions, pot experiments were performed according to Hufnagel et al. [29]. Generally, the sorghum seeds were sown in plastic pots in a greenhouse in the experimental station of Nanjing Agricultural University in 2015 and 2016. The Olsen-P of soil used in the experiments was 1.25 μg/g. After 7 days, two accessions were allowed to continue growing under sufficient-P (30 μg/g) and low-P (3 μg/g) conditions in each pot for 40 days.

The roots of sorghum were harvested and cleaned carefully after breaking the pots. Subsequently, electronic images of root morphology were captured using an EPSON scanner and analyzed by WinRHIZO software. Roots and shoots were heated at 105 °C for 30 min and then oven-dried at 65 °C for 72 h. The samples were weighed and milled. A portion of the milled samples was digested with HNO_3_. The total P content of shoots was determined by ICP-OES (PerkinElmer, Optima 8000, USA). The relative length of roots (RLR) was expressed by the ratio of the root length of plants treated with low P to that treated with sufficient P. The relative surface area of root (RSAR), relative average diameter of root (RADR), relative volume of root (RVR), relative number of root tips (RNRT), relative dry weight of root (RDWR), relative dry weight of shoot (RDWS) and relative P content of shoot (RPCS) were calculated using similar methods.

### Hydroponic experiments

To determine the duration of the low-P treatment and facilitate the harvest of roots for RNA-seq, hydroponic experiments were performed according to Hufnagel et al. [29]. Sorghum seeds were sterilized with 75% ethanol for 5 min, dried, and then germinated in moistened filter paper. After 3 days, seedlings with similar growth vigor were transplanted into distilled water. When the seedlings reached the three-leaf stage, they were subjected to sufficient-P conditions (Hoagland (5 mmol/L Ca(NO_3_)_2_·4H_2_O, 5 mmol/L KNO_3_, 2 mmol/L MgSO_4_·7H_2_O, 0.025 mmol/L Fe-EDTA, 1 μmol/LH_3_BO_3_, 1 μmol/L MnSO_4_·H_2_O, 1 μmol/L ZnSO_4_·7H_2_O, 0.5 μmol/L CuSO_4_·5H_2_O, and 0.005 μmol/L (NH_4_)_6_Mo_7_O_24_·4H_2_O) with 1.0 mmol/L KH_2_PO_4_) and low-P conditions (Hoagland with 1 μmol/L KH_2_PO_4_ and 1.0 mmol/L KCl) for 2 weeks. The nutrient solution with a pH of 5.8 ± 0.1 was replaced every 3 days. The seedlings were cultured in a growth chamber under a 12 h/12 h (day/night) photoperiod and a temperature cycle of 28 °C/22 °C (day/night). Each treatment was replicated three times. Roots and shoots of plants subjected to treatment with different P concentrations (sufficient-P and low-P conditions) were harvested every 2 days and used for measuring the ratio of root dry weight to shoot dry weight (R/S ratio) and gene expression analysis.

### RNA isolation and transcriptome sequencing

Total RNA was isolated using TRIZOL (Invitrogen, USA) from the roots of accessions 12484 and 13443 harvested after 8 days of P treatment. The quality and integrity of the RNA were checked by an Agilent Bioanalyzer 2100 system (Agilent Technologies, CA, USA). The total RNA concentration was assessed using a QUBIT RNA ASSAY KIT (Invitrogen, USA). mRNA was enriched by the NEBNext® Poly(A) mRNA Magnetic Isolation Module (Invitrogen, USA), and the mRNA molecules were fragmented and subsequently used in first- and second-strand cDNA syntheses. The cDNA was subsequently subjected to terminal repair and poly (A) and unique adapter ligation. Prior to sequencing, the cDNA fragments were amplified and purified. The purified amplification products were sequenced on an Illumina Hiseq 2500 platform (CapitalBio Technology, Beijing, China).

### RNA-seq data analysis

The original sequencing data were defined as raw reads. The clean reads were generated from the raw reads after removing the low-quality reads, mismatches, and adaptor sequences. The sequences of the clean reads were aligned with the sorghum transcript sequence (Ensembl, http://plants.ensembl.org/index.html). The perfectly matching sequences were used for further analyses. The gene expression levels were normalized by fragments per kilo base of transcript per million fragments mapped (FPKM) and analyzed using Cufflinks software (http://cole-trapnell-lab.github.io/cufflinks/) [40, 41]. DEGs were analyzed using Cuffdiff software [42]. The DEGs were identified based on the following criteria: the absolute value of the Log fold change had to be ≥1, the *P* value adjusted using the false discovery rate (FDR) method had to be ≤0.05, and three biological replicates had to be used.

DEGs were subjected to GO analyses, as follows: 1) all DEGs were mapped to GO terms in the database (http://www.geneontology.org/); 2) the gene numbers of each term were calculated; 3) the hypergeometric test was used to analyze the significantly enriched GO terms in the DEGs; 4) the input frequency represented the ratio of the number of DEGs with annotation to the total number of DEGs, and the background frequency represented the ratio of the number of genes with annotation to the total number of genes.

To analyze the pathways that were significantly associated with DEGs, we used the same method to blast the DEGs with the KEGG database (http://www.genome.jp/kegg/pathway.html), which is a major public pathway-related database. Then, the gene numbers of each pathway were calculated, and the hypergeometric test was used to analyze the significantly enriched pathways.

### Quantitative real-time polymerase chain reaction (qRT-PCR)

Total RNA was extracted from each sample by using the Plant RNA Extract Kit (TIANGEN, Beijing, China), and approximately 0.5 μg of total RNA was used for cDNA synthesis using HiScript® II Q RT SuperMix for qPCR (+gDNA wiper) (Vazyme, Nanjing, China). After diluting the cDNA reaction mixture five times, 2 μL of the reaction mixture was used as template in a 20-μL reaction system. In addition, the reaction system contained 0.8 μL of 10 μmol/L gene-specific primers (Additional file 1: Table S1) and 10 μL of AceQ® qPCR SYBR® Green Master Mix (Low ROX Premixed) (Vazyme, Nanjing, China). qRT-PCR was performed on an ABI 7500 real-time PCR system (Applied Biosystems, Forster City, CA, USA), and the data were analyzed using the ABI 7500 Sequence Detection System software v.1.4. A sorghum constitutive expression gene, *18S rRNA,* was used as the reference gene for normalization. Three technical replicates were carried out for each sample.

### Malate application experiments

Accession 13443 was used as the experimental material. Hydroponic experiments were performed according to Mora-Macías et al. [43]. After seeds germinated in distilled water for 1 week, the seedlings displaying similar growth vigor were picked and subjected to sufficient P (1.0 mmol/L KH_2_PO_4_) with and without 1.0 mmol/L malate or low P (1.0 μmol/L KH_2_PO_4_) with and without malate for 2 weeks. Each treatment was replicated three times. The lengths of the primary root and root tips were measured.

## Results

### Identification of the low-P-tolerant accession (12484) and the low-P-sensitive accession (13443)

**Fig. 1:**
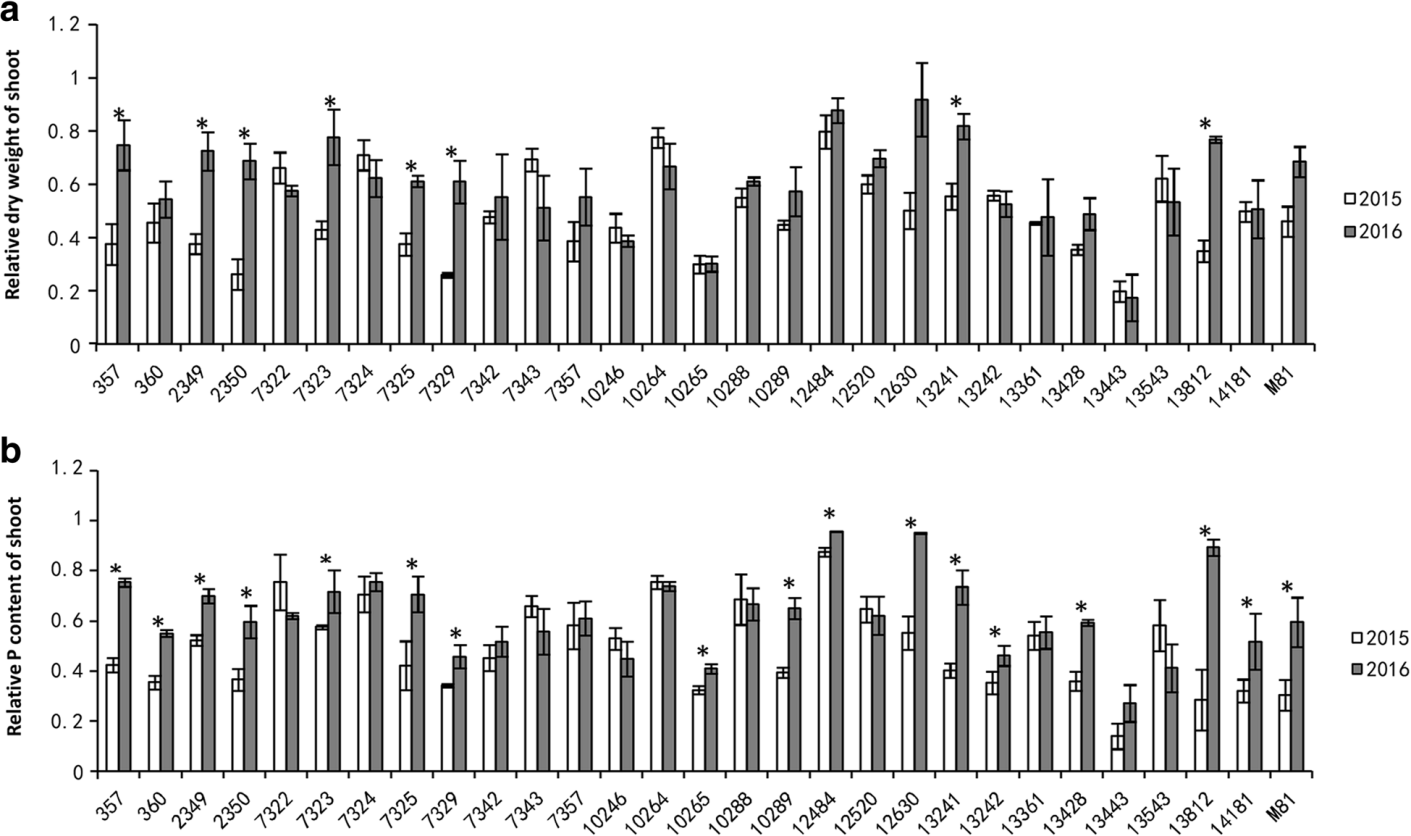
Physiological changes of sorghum accessions in response to low-P stress. **a** and **b** Relative dry weight of shoot (**a**) and relative P content of shoot (**b**) of different accessions from 2015 and 2016 experiments. Asterisks denote statistically significant differences between the results from two independent experiments (*P* < 0.05). Bars represent the standard error of the mean (*n* = 3)

**Fig. 2:**
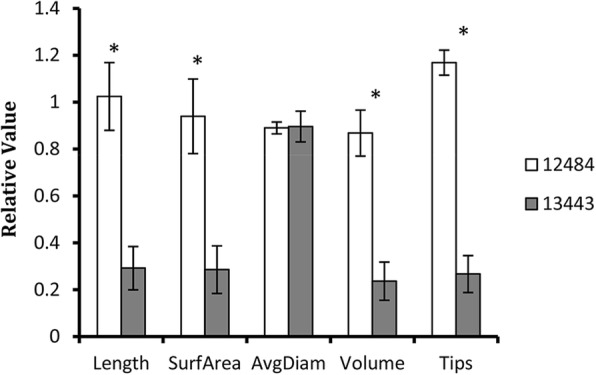
Effect of low-P stress on root morphologies of sorghum accessions 12484 and 13443 grown in soil. Asterisks denote statistically significant differences between the two accessions (P < 0.05) by Student’s t-test. Bars represent the standard error of the mean (n = 3)

It is well documented that the biomass of the shoot is greatly reduced by P-deficient treatment [44, 45], and shoot biomass is a good indicator of plant response to P deficiency tolerance. We expected that the shoot biomass of the low-P-tolerant accession would be less negatively affected by low-P treatment than by sufficient-P treatment; thus, the low-P-tolerant accession would display a high RDWS, and vice versa for the low-P-sensitive accession. A total of 29 sorghum accessions were grown in both low-P and sufficient-P conditions. These accessions displayed great variability in RDWS with values ranging from 0.2 to 0.9. The RDWS results were generally consistent between two independent experiments carried out in two consecutive years (2015 and 2016). Among them, accession 12484 displayed the highest RDWS, while accession 13443 showed the lowest values in both years (Fig. 1). Therefore, we selected accessions 12484 and 13443 as the low-P-tolerant and low-P-sensitive accessions, respectively, for further analysis. Meanwhile, the root morphology of each accession was tested. The RPCS showed a significant positive correlation with RDWS, and there were significant positive correlations with RDWR, RNRT, RSAR, RLR, and RVR (Table 1). Moreover, the differences in RNRT, RSAR, RLR and RVR between the selected accessions 12484 and 13443 were significant (Fig. 2).

**Table 1:** Correlation analysis between root morphology and P starvation tolerance. Asterisks note statistically significant differences between control and treatment groups (P < 0.05), double asterisks note statistically significant differences between control and treatment groups (P < 0.01). Bars represent the standard error of the mean (n = 3)

2015/2016	Relative length of root	Relative surface area of root	Relative average diam of root	Relative volume of root	Relative number of root tips	Relative dry weight of root	Relative dry weight of shoot	Relative P content of shoot
Relative length of root	1	0.893^**^/0.948^**^	0.101/0.137	0.719^**^/0.812^**^	0.918^**^/0.840^**^	0.969^**^/0.422^**^	0.539^**^/0.475^**^	0.572^**^/0.507^**^
Relative surface area of root	0.893^**^/0.948^**^	1	0.486^**^/0.435^**^	0.953^**^/0.954^**^	0.695^**^/0.694^**^	0.977^**^/0.529^**^	0.591^**^/0.552^**^	0.521^**^/0.578^**^
Relative average diam of root	0.101/0.137	0.486^**^/0.435^**^	1	0.678^**^/0.665^**^	−0.129/−0.208	0.315/0.444^*^	0.429^*^/0.360	0.138/0.342
Relative volume of root	0.719^**^/0.812^**^	0.953^**^/0.954^**^	0.678^**^/0.665^**^	1	0.468^**^/0.488^**^	0.868^**^/0.587^**^	0.543^**^/0.567^**^	0.426^**^/0.596^**^
Relative number of root tips	0.918^**^/0.840^**^	0.695^**^/0.694^**^	−0.129/−0.208	0.468^**^/0.488^**^	1	0.821^**^/0.405^*^	0.485^**^/0.495^**^	0.542^**^/0.524^**^
Relative dry weight of root	0.969^**^/0.422^**^	0.977^**^/0.529^**^	0.315/0.444^*^	0.868^**^/0.587^**^	0.821^**^/0.405^*^	1	0.582^**^/0.647^**^	0.560^**^/0.687^**^
Relative dry weight of shoot	0.539^**^/0.475^**^	0.591^**^/0.552^**^	0.429^*^/0.360	0.543^**^/0.567^**^	0.485^**^/0.495^**^	0.582^**^/0.647^**^	1	0.797^**^/0.876^**^
Relative P content of shoot	0.572^**^/0.507^**^	0.521^**^/0.578^**^	0.138/0.342	0.426^**^/0.596^**^	0.542^**^/0.524^**^	0.560^**^/0.687^**^	0.797^**^/0.876^**^	1

* menas *P* < 0.05; ** means *P* < 0.01

### Increased P content in the shoot accounts for enhanced tolerance to low P

Plants can achieve tolerance to low P availability by optimizing the P utilization and/or by improving P acquisition from the soil [46]. To understand whether the variations in low-P tolerance of the 29 sorghum accessions were due to their improved ability to acquire P, we determined the P content in the shoot tissues of these accessions grown under both low-P and sufficient-P conditions. Figure 1b shows that low-P-tolerant accessions displayed high P content, while sensitive accessions displayed low P content. In general, there was a good correlation between RPCS and RDWS, with correlation coefficients equal to 0.797 and 0.876 in 2015 and 2016, respectively, indicating variation in P acquisition ability accounts for the variation in low-P tolerance in these selected sorghum accessions.

### Determining optimal sampling time for RNA-seq

**Fig. 3:**
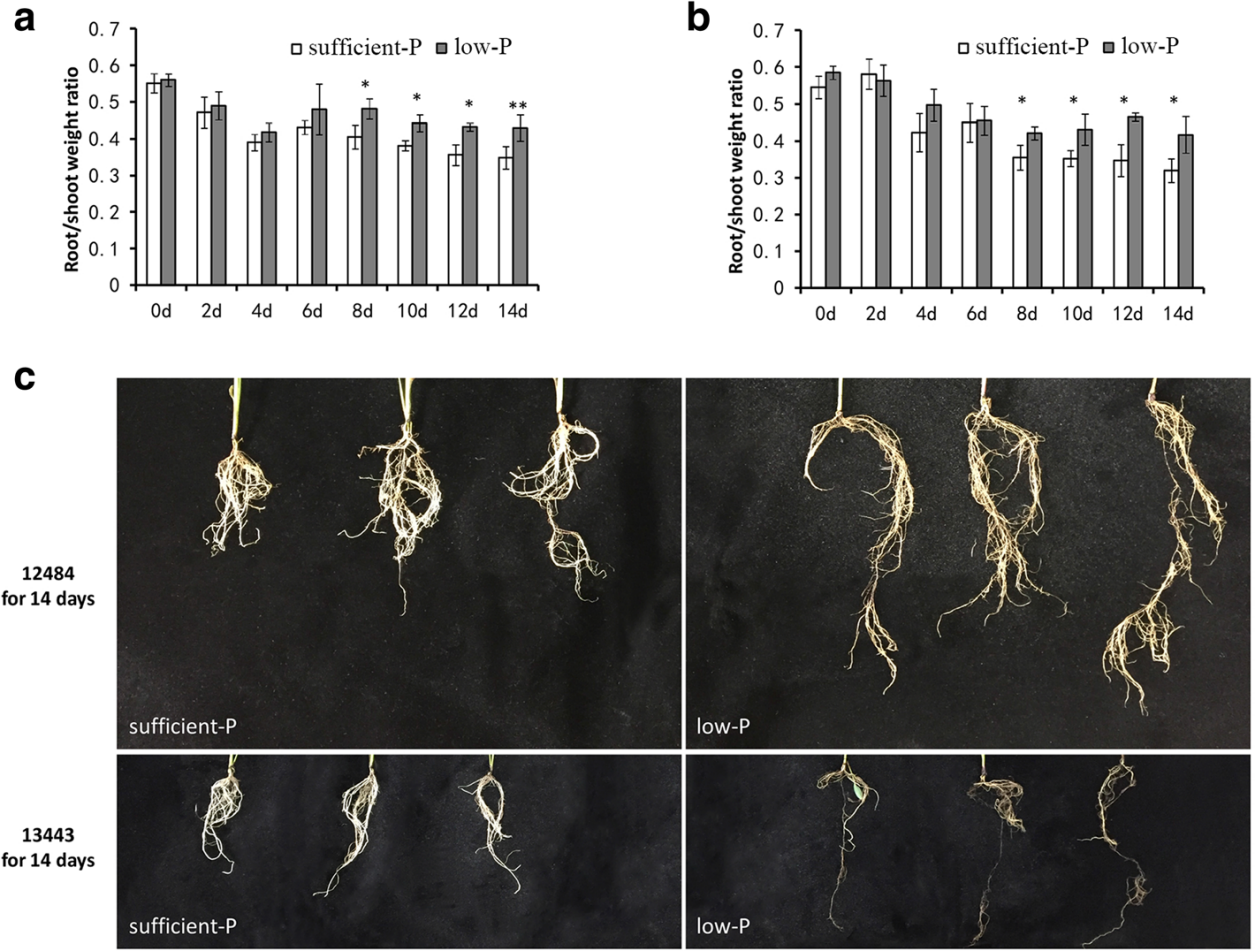
Physiological responses of sorghum accessions 12484 and 13443 to low-P stress in the hydroponic system. **a** and **b** Root/shoot dry weight ratio of accession 12484 (**a**) and accession 13443 (**b**). **c** Photographs of representative plants. Asterisks denote significant differences between control and treatment groups by Student’s t-test (* means P < 0.05; ** means *P* < 0.01). Bars represent the standard error of the mean (n = 3)

To analyze the transcript profiles of low-P-tolerant and -sensitive accessions in responding to low P, we first determined the optimal sampling time by analyzing the R/S of sorghum grown hydroponically under low P or sufficient P supply. An increasing R/S ratio is a hallmark indicator of plant responses to P-deficient conditions [44, 47]. The R/S ratio of accession 12484 seedlings grown under low-P conditions was not significantly different than that grown under sufficient-P conditions after six successive days of treatment (Fig. 3). In contrast, from the 8th to the 14th day after low-P treatment, the R/S ratio was significantly higher than that of plants grown under sufficient-P conditions. Moreover, a similar trend was found in accession 13443. Therefore, the roots of seedlings grown under low P and sufficient P for 8 days were sampled for RNA-seq analysis.

### RNA-seq analysis and de novo assembly

The libraries from the roots of accessions 12484 and 13443 grown under low-P and sufficient-P conditions for 8 days were sequenced using Illumina high-throughput sequencing technology. Approximately 512 million raw reads were generated, and 492 million clean reads were obtained after cleaning and quality checks (Table 2). The clean rates and the Q20 and Q30 values of the samples reached up to 96, 97, and 93%, respectively, indicating the high quality of the sequencing results. Approximately 87% of the total clean reads were mapped to the sorghum reference sequence Sorghum_bicolor.Sorbi1.33 (Ensembl; http://plants.ensembl.org/index.html), > 97.5% of which were uniquely mapped reads.

**Table 2:** Summary of RNA-seq data and de novo assembly

accession	Sample	Raw reads number	Clean reads number	Clean rate(%)	Q20(%)	Q30(%)	Mapped reads rate(%)	Uniquely mapped reads rate(%)	Gene Number	Transcript Number
13443	sufficient P_1	44920566	43070114	95.88	97.62	93.31	89.36	97.45	25489	33223
	sufficient P_2	42063644	40616024	96.56	97.76	93.63	85.21	97.43	26110	33633
	sufficient P_3	30906152	29804314	96.43	97.73	93.55	88.59	97.54	24579	31528
	low P_2	37808338	36157312	95.63	97.6	93.28	88.36	97.62	24765	31741
	low P_3	48044802	46225956	96.21	97.71	93.54	87.75	97.66	25942	33394
	low P_5	51611204	49267030	95.46	97.54	93.16	89.32	97.41	25331	33283
12484	sufficient P_1	41036008	39463806	96.17	97.69	93.48	86.76	97.46	25018	32024
	sufficient P_2	44683368	42943164	96.11	97.71	93.56	84.96	97.37	25196	32012
	sufficient P_3	43627358	41878828	95.99	97.67	93.46	84.02	97.46	25308	32157
	low P_1	41144016	39708924	96.51	97.76	93.63	88.35	97.55	24587	31120
	low P_2	46931232	45299694	96.52	97.75	93.61	86.93	97.52	24935	31395
	low P_5	39105304	37577298	96.09	97.65	93.39	87.27	97.61	24670	31545

### Screening of the candidate genes related to P starvation tolerance in sorghum

**Fig. 4:**
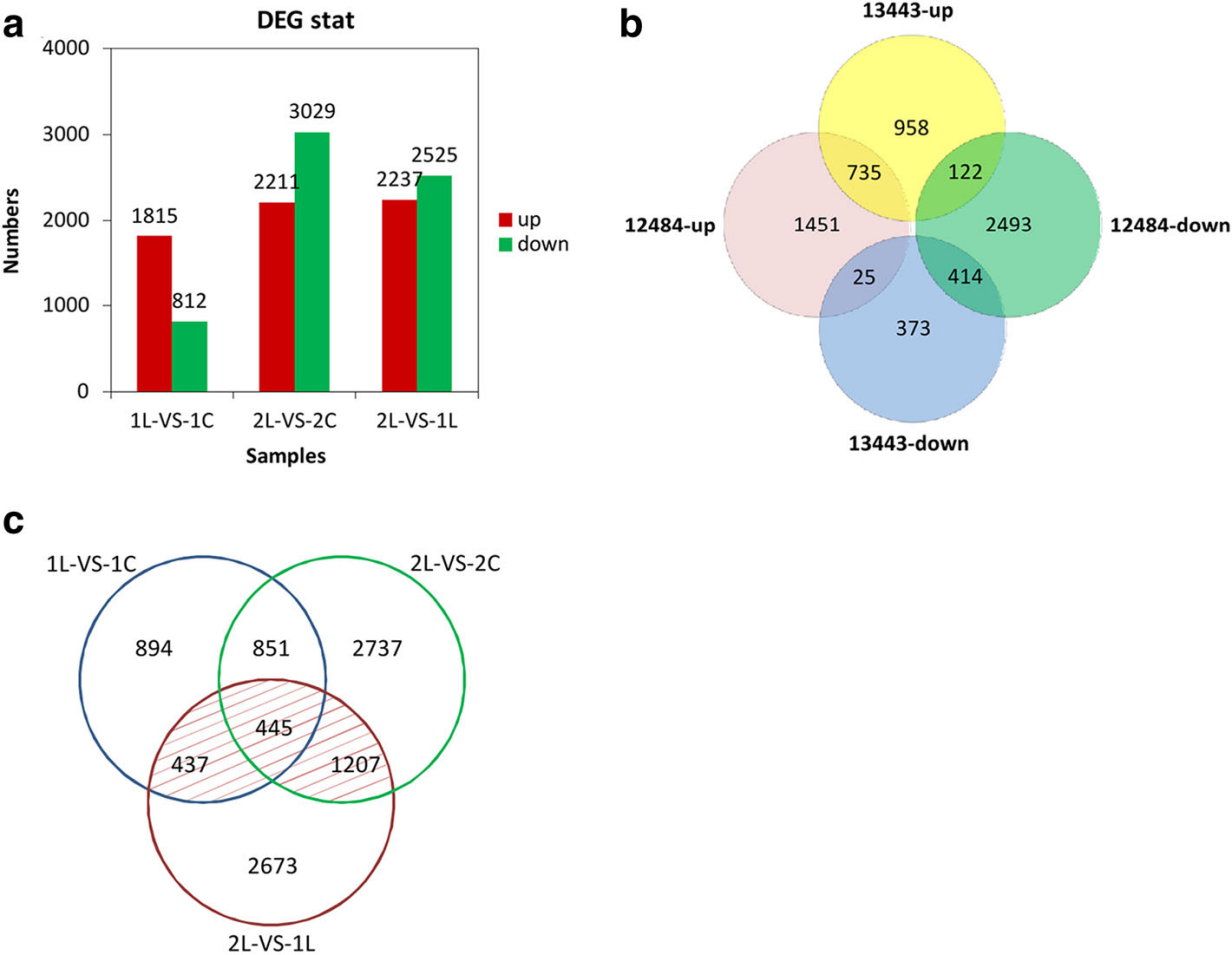
Comparison of the number of DEGs. 1: low-P-sensitive accession 13443; 2: low-P-tolerant accession 12484; L: low P; C: sufficient P. **a** The number of differentially expressed genes in each part. **b** Venn diagram illustrating the genes of the two accessions in response to low-P stress. **c** Response of DEGs to low-P stress and different accessions under low-P conditions

Comparing the transcripts of sorghum grown under low-P conditions with those under sufficient-P conditions, a total of 2627 DEGs in accession 13443 and 5240 DEGs in accession 12484 were identified. Among these DEGs, 1815 were upregulated and 812 were downregulated in accession 13443, while 2211 and 3029 DEGs were upregulated and downregulated in accession 12484, respectively (Fig. 4a). Moreover, 735 DEGs were upregulated and 414 DEGs were downregulated in both accessions (Fig. 4b). Interestingly, the numbers of DEGs, regardless of their upregulation or downregulation, in accession 13443 were lower than those in accession 12484. Here, the 5275 nonredundant DEGs and the 1296 overlapping DEGs, for a total of 6571 DEGs, were identified as DEGs in response to low-P stress in sorghum.

A total of 4762 nonredundant DEGs, of which 2237 were upregulated and 2525 were downregulated, were identified in the low-P-tolerant accession 12484 vs. the low-P-sensitive accession 13443 under low-P conditions (Fig. 4a). During our study, these 4762 nonredundant DEGs were identified as DEGs in different accessions under low-P conditions.

Finally, comparing these two groups, a total of 2089 common DEGs were found (Fig. 4c), meaning these genes were not only differentially expressed in response to low-P stress but also differentially expressed in different accessions under low-P stress. These DEGs were identified as the candidate genes related to P starvation tolerance in sorghum.

### qRT-PCR verification

**Fig. 5:**
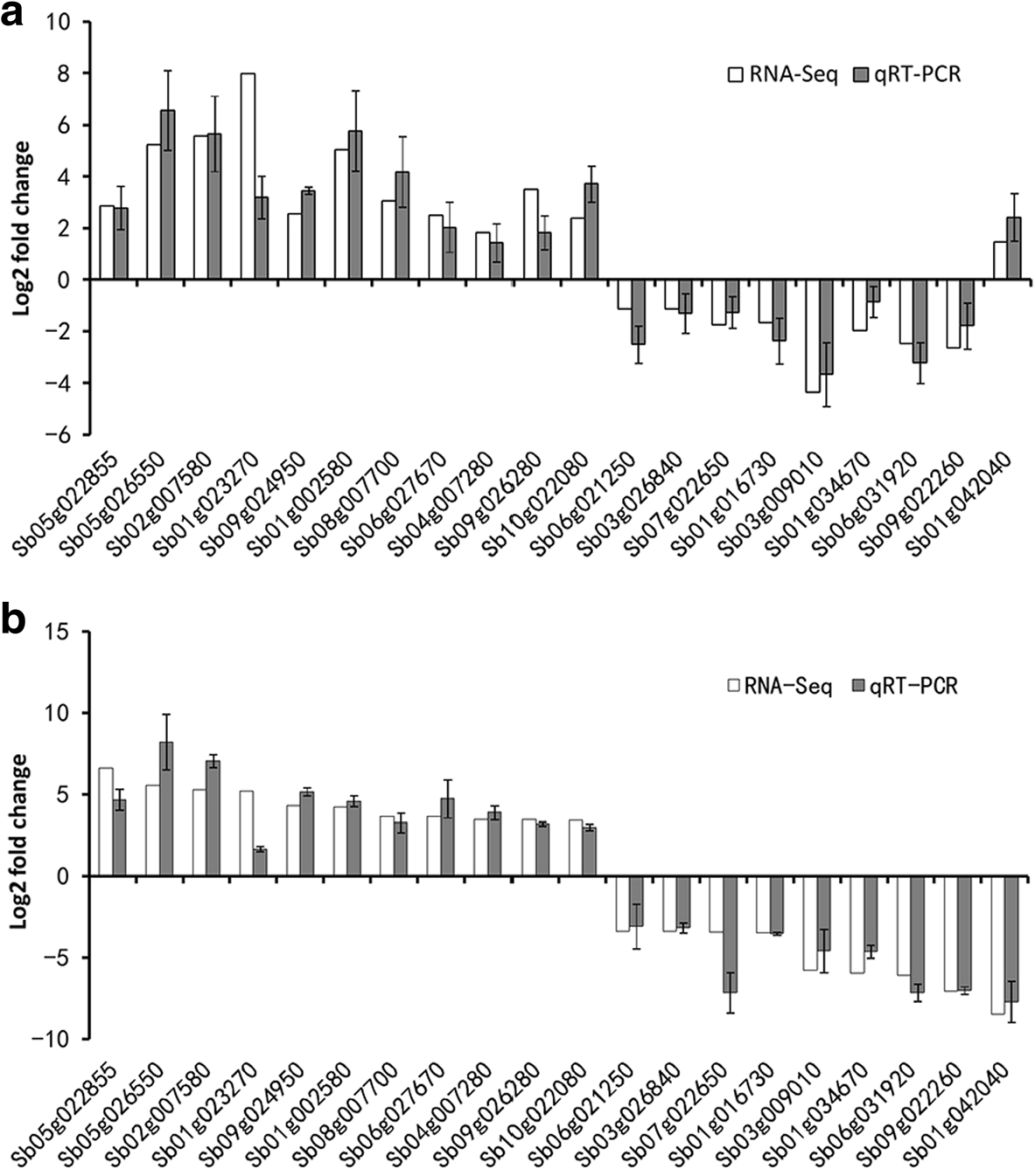
Validation of transcript abundance obtained from RNA-seq using qRT-PCR. Twenty randomly chosen genes were used for validation. **a** and **b** Relative expression by RNA-seq and qRT-PCR of accession 13443 (**a**) and accession 12484 (**b**). Bars represent the standard error of the mean (n = 3)

To verify the RNA-seq results, we determined the abundance of 20 randomly selected DEGs by qRT-PCR assay. The qRT-PCR assay showed that *Sb05g022855*, *Sb05g026550*, *Sb02g007580*, *Sb01g023270*, *Sb09g024950*, *Sb01g002580*, *Sb08g007700*, *Sb06g027670*, *Sb04g007280*, *Sb09g026280* and *Sb10g022080* were upregulated, whereas *Sb06g021250*, *Sb03g026840*, *Sb07g022650*, *Sb01g016730*, *Sb03g009010*, *Sb01g034670*, *Sb06g031920* and *Sb09g022260* were downregulated in both accessions (Fig. 5). Additionally, *Sb01g042040* was downregulated in accession 12848 but was upregulated in accession 13443. These results were consistent with the data obtained from the RNA-seq analysis, indicating the reliability of the RNA-seq results.

### Annotation and function classification of DEGs

To evaluate the potential functions of those DEGs, GO analyses were performed on the DEGs identified during each comparison. Among the DEGs identified in response to low-P stress, 1578 of the 2627 DEGs in accession 13443 were assigned to at least one GO term, whereas 3147 of the 5240 DEGs in accession 12484 were assigned to at least one GO term. Among the DEGs in different accessions under low-P conditions, 2317 of the 4762 DEGs were assigned to different GO terms. Among the candidate DEGs, 1006 of the 2089 DEGs were assigned to at least one GO term.

To deeply investigate the functions of DEGs, GO enrichment of each group was analyzed, and the top 30 GO enrichment terms were listed (Additional file 2: Figure S1). The results showed the following: 1) For the DEGs in the different accessions under low-P conditions, many of the top 30 significantly enriched GO terms were in the molecular function category, suggesting these two accessions had significant differences in molecular functions under low-P conditions. 2) Candidate genes were significantly enriched in the molecular function and biological process categories, implying that molecular functions and biological processes play important roles in P starvation tolerance of sorghum. Within the molecular function category, GO terms related to oxidoreductase activity (GO:0016491, GO:0016705, GO:0016684, GO:0051213, GO:0004601, GO:0004497) were significantly enriched.

The candidate genes enriched in GO terms for which the Input frequency/Background frequency ratio was higher than 1 were examined and are listed in Table 3. The results showed that a total of 20 GO terms exhibited a higher Input frequency than Background frequency, suggesting that the expression changes of these genes might affect the functions of these GO terms. Among them, GO terms related to antioxidant activity, transporter activity, nucleic acid binding TF activity, catalytic activity, extracellular region, membrane part and response to stimulus also showed a higher Input frequency than Background frequency in the DEGs in response to low-P stress and the DEGs in different accessions under low-P conditions.

**Table 3:** List of the focused significantly enrichedment GO terms

Namespace	ID	Term	Input frequency/Background frequency			
			13443(L/C)	12484(L/C)	12484 L vs 13443 L	candidate genes
molecular function	GO:0016209	antioxidant activity	1.9422	2.0953	1.8839	2.6634
molecular function	GO:0005215	transporter activity	1.2064	1.3471	1.2791	1.3071
molecular function	GO:0001071	nucleic acid binding transcription factor activity	1.3734	1.3157	1.2425	1.2459
molecular function	GO:0003824	catalytic activity	1.1786	1.1433	1.1391	1.2145
cellular component	GO:0005576	extracellular region	1.1444	1.8283	1.7582	2.4556
cellular component	GO:0044425	membrane part	1.3049	1.2736	1.0791	1.0349
biological process	GO:0050896	response to stimulus	1.0837	1.1235	1.0924	1.0360
molecular function	GO:0060089	molecular transducer activity	0.8920	1.0287	1.0935	1.2583
molecular function	GO:0030234	enzyme regulator activity	0.8054	1.0453	0.8389	1.1622
molecular function	GO:0009055	electron carrier activity	1.0321	0.9704	0.9665	1.1497
molecular function	GO:0098772	molecular function regulator	0.8312	1.0748	0.8044	1.1363
molecular function	GO:0045735	nutrient reservoir activity	0.4629	1.7021	0.9457	1.1098
cellular component	GO:0016020	membrane	1.1236	1.1582	0.9954	1.0698
biological process	GO:0001906	cell killing	–	–	–	2.9594
biological process	GO:0048511	rhythmic process	0.5311	1.3314	1.6275	2.4213
biological process	GO:0040011	locomotion	1.1418	1.0370	–	1.6646
biological process	GO:0051704	multi-organism process	0.9849	1.0975	0.9937	1.5259
biological process	GO:0044699	single-organism process	1.0054	1.0551	0.9164	1.1612
biological process	GO:0008152	metabolic process	0.9470	0.9064	0.8909	1.1021
biological process	GO:0002376	immune system process	0.7807	1.1255	1.1963	1.0871

“—” means GO term not significantly enreached

### Significant enrichment pathways

Genes usually play roles in certain biological processes by interacting with each other. Therefore, pathway analyses are helpful for further understanding the biological functions of genes. Pathway enrichment analysis of each group’s DEGs based on the KEGG database was performed, and the significantly enriched pathways are listed in Table 4. Notably, almost all of the significantly enriched pathways were involved in metabolism.

**Table 4:** List of significantly enrichedment pathways

Term	Pathways Identifiers	*P*-Value				First class	Second class
		13443 (L/C)	12484 (L/C)	12484 L vs 13443 L	candidate DEGs		
Phenylpropanoid biosynthesis	map00940	4.2902E-06	6.0067E-06	0.0001	6.3352E-09	Metabolism	Biosynthesis of other secondary metabolites
Phenylalanine metabolism	map00360	2.5179E-05	4.1713E-06	0.0010	1.2791E-06	Metabolism	Amino acid metabolism
Biosynthesis of secondary metabolites	map01110	0.0002	0.0001	0.0001	2.2959E-06	Metabolism	Global and overview maps
Metabolic pathways	map01100	–	0.0023	0.0084	0.0068	Metabolism	Global and overview maps
Flavone and flavonol biosynthesis	map00944	0.0278	–	0.0244	0.0116	Metabolism	Biosynthesis of other secondary metabolites
Carotenoid biosynthesis	map00906	–	0.0038	0.0188	0.0189	Metabolism	Metabolism of terpenoids and polyketides
Starch and sucrose metabolism	map00500	–	0.0010	–	0.0217	Metabolism	Carbohydrate metabolism
Monoterpenoid biosynthesis	map00902	–	0.0218	–	0.0223	Metabolism	Metabolism of terpenoids and polyketides
Galactose metabolism	map00052	–	0.0168	0.0056	0.0368	Metabolism	Carbohydrate metabolism
Flavonoid biosynthesis	map00941	–	0.0398	–	0.0403	Metabolism	Biosynthesis of other secondary metabolites
Nitrogen metabolism	map00910	–	0.0359	–	0.0483	Metabolism	Energy metabolism
Glutathione metabolism	map00480	–	0.0016	–	–	Metabolism	Metabolism of other amino acids
Amino sugar and nucleotide sugar metabolism	map00520	–	0.0045	–	–	Metabolism	Carbohydrate metabolism
Plant hormone signal transduction	map04075	–	0.0082	–	–	Environmental Information Processing	Signal transduction
alpha-Linolenic acid metabolism	map00592	–	0.0250	–	–	Metabolism	Lipid metabolism
Zeatin biosynthesis	map00908	–	0.0302	–	–	Metabolism	Metabolism of terpenoids and polyketides
Glycerolipid metabolism	map00561	0.0004	–	–	–	Metabolism	Lipid metabolism
Cutin, suberine and wax biosynthesis	map00073	0.0066	–	–	–	Metabolism	Lipid metabolism
Phenylalanine, tyrosine and tryptophan biosynthesis	map00400	0.0213	–	–	–	Metabolism	Amino acid metabolism
Taurine and hypotaurine metabolism	map00430	0.0277	–	–	–	Metabolism	Metabolism of other amino acids
Degradation of aromatic compounds	map01220	0.0278	–	–	–	Metabolism	Global and overview maps
Stilbenoid, diarylheptanoid and gingerol biosynthesis	map00945	0.0362	–	–	–	Metabolism	Biosynthesis of other secondary metabolites
Glycerophospholipid metabolism	map00564	0.0481	–	–	–	Metabolism	Lipid metabolism
Cysteine and methionine metabolism	map00270	–	–	0.0187	–	Metabolism	Amino acid metabolism
Thiamine metabolism	map00730	–	–	0.0260	–	Metabolism	Metabolism of cofactors and vitamins

“—” means that the P-Value is greater than 0.05

For those pathways, 1) 3 pathways (phenylpropanoid biosynthesis, phenylalanine metabolism, and biosynthesis of secondary metabolites) were significantly enriched by low-P treatment in both accessions; 2) pathways specifically enriched in accession 12484 by low-P stress include sugar metabolism (starch and sucrose, galactose, amino sugar and nucleotide sugar) and carotenoid biosynthesis, while those in accession 13443 include amino acid metabolism (phenylalanine, tyrosine, tryptophan, taurine and hypotaurine) and lipid metabolism (glycerolipid and glycerophospholipid); 3) for the candidate DEGs, 11 pathways were significantly enriched, and most of them were only significantly enriched in accession 12484 in response to low-P stress.

### Candidate genes involved in plant hormone signal transduction

Almost all of the significantly enriched pathways were involved in metabolism. Interestingly, only one term (plant hormone signal transduction) involved in signal transduction was from the class of environmental information processing (Table 4). Recent evidence has proven that hormones participate in the control of plants in response to P starvation [48, 49]. Therefore, the DEGs related to plant hormone signal transduction were chosen for further analysis.

**Fig. 6:**
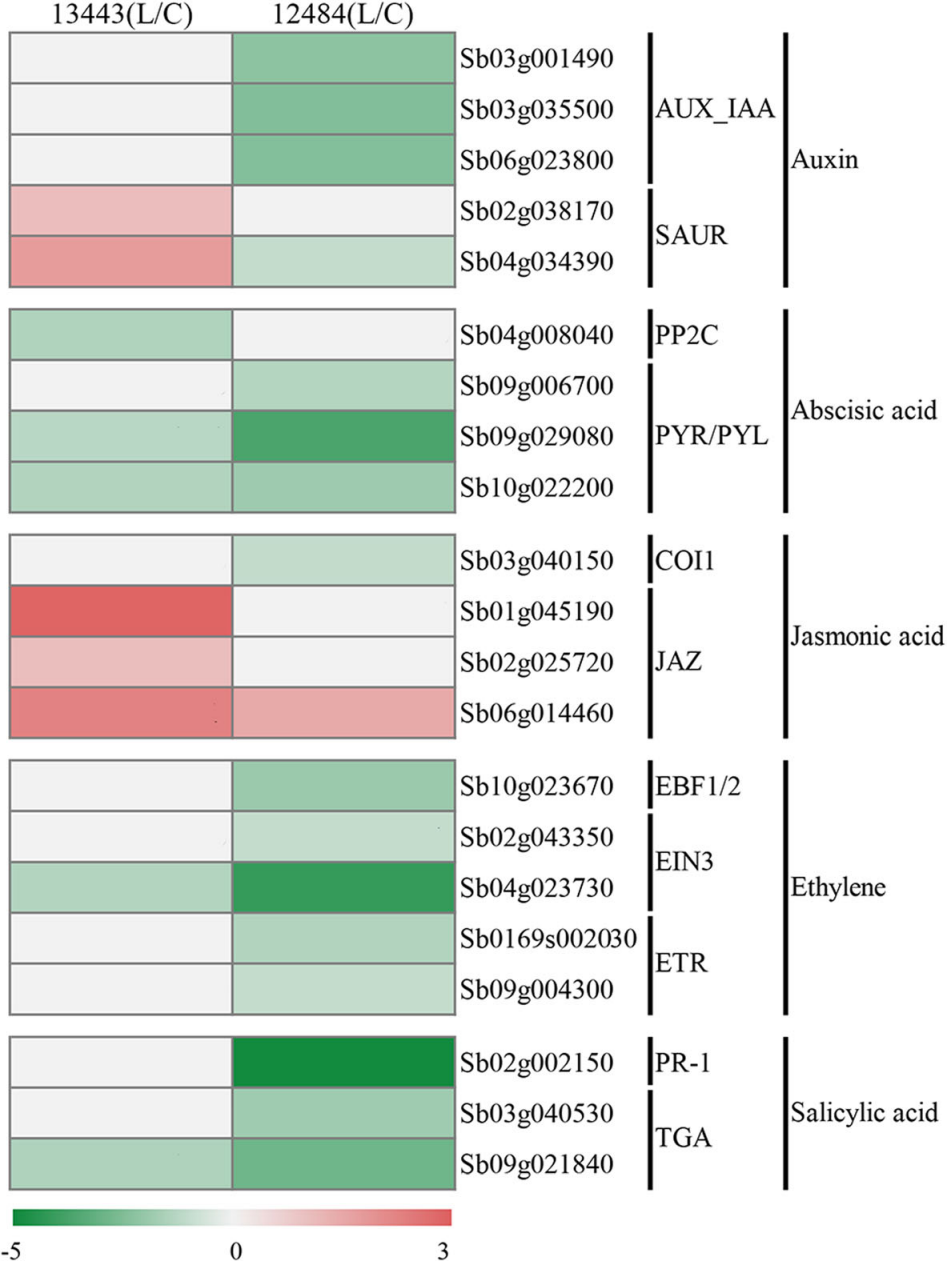
Heatmap of the candidate genes involved in plant hormone signal transduction. The log2 fold change of the candidate genes involved in plant hormone signal transduction under low-P conditions compared with that under sufficient-P conditions in each section is represented by a color scale consisting of red (upregulated), white (not regulated) and green (downregulated)

A total of 21 candidate DEGs were found to be associated with plant hormone (including auxin (AUX), abscisic acid (ABA), jasmonic acid (JA), ethylene (ET), and salicylic acid (SA)) signal transduction (Fig. 6). Among them, five DEGs were AUX signaling pathway-related genes, including two *small auxin upregulated RNA* (*SAUR*) genes and three *Aux/IAA* genes. Moreover, four candidate genes were ABA signaling pathway-related genes, including one *protein phosphatase 2C* (*PP2C*) and three *PYR/PYL* genes. Furthermore, four candidate genes were involved in the JA signaling pathway, including three *jasmonatezim-domain* (*JAZ*) genes and one *coronatine insensitive 1* (*COI1*) gene. In addition, there were seven candidate genes related to the ET signaling pathway, including one *EBF1/2* gene, two *EIN3* genes, two Ethylene-insensitive-3-like (EIL) genes and two *ETR* genes, and three candidate genes related to the SA signaling pathway, including one *PR-1* gene and two *TGA* genes.

### Transcriptional factors (TFs) identified from candidate genes

A total of 127 nonredundant TFs were identified from the candidate DEGs using the plant TF database PlantTFDB version 4.0 (http://planttfdb.cbi.pku.edu.cn/). In general, 109 were responding to low P in accession 12848, while 50 were responding to low P in accession 13443. Among them, bHLH, WRKY and MYB were the top three most abundant TFs, numbering 17, 13, and 12, respectively (Table 5).

**Table 5:** List of genes belonging to TFs

TF-Family	Gene	Expression (in response to low P)		TF-Family	Gene	Expression (in response to low P)	
		13443	12484			13443	12484
AP2	Sb01g003400	up	up	HD-ZIP	Sb01g022420	–	up
B3	Sb09g004870	–	up	HD-ZIP	Sb06g025750	–	up
bHLH	Sb03g008290	–	up	HD-ZIP	Sb04g023410	–	down
bHLH	Sb10g005650	–	up	HD-ZIP	Sb06g024000	–	down
bHLH	Sb02g027210	–	up	HSF	Sb01g021490	up	up
bHLH	Sb01g045710	–	up	HSF	Sb01g008380	up	–
bHLH	Sb01g043570	–	up	HSF	Sb03g033750	–	down
bHLH	Sb03g036100	up	up	LSD	Sb07g004050	–	up
bHLH	Sb07g004190	up	up	LBD	Sb09g030780	up	–
bHLH	Sb2250s002010	up	–	LBD	Sb01g014800	–	down
bHLH	Sb09g006220	up	–	MIKC_MADS	Sb07g001250	–	up
bHLH	Sb04g017390	down	down	MIKC_MADS	Sb06g017660	–	up
bHLH	Sb04g027280	–	down	MYB	Sb03g004600	–	up
bHLH	Sb01g041960	–	down	MYB	Sb03g032260	–	up
bHLH	Sb03g042860	–	down	MYB	Sb02g040480	–	up
bHLH	Sb05g023730	–	down	MYB	Sb08g016620	up	–
bHLH	Sb06g028750	–	down	MYB	Sb02g030900	up	–
bHLH	Sb06g020810	–	down	MYB	Sb08g018840	down	up
bHLH	Sb03g005250	–	down	MYB	Sb03g003120	down	down
bZIP	Sb08g020600	–	up	MYB	Sb09g001590	–	down
bZIP	Sb04g008840	–	up	MYB	Sb03g012310	–	down
bZIP	Sb04g007060	–	up	MYB	Sb02g024260	–	down
bZIP	Sb07g025490	–	up	MYB	Sb02g043420	–	down
bZIP	Sb01g037520	up	up	MYB	Sb09g002680	–	down
bZIP	Sb04g000300	up	–	MYB_related	Sb09g029560	–	up
bZIP	Sb09g021840	down	down	MYB_related	Sb03g006440	–	down
bZIP	Sb09g024290	–	down	MYB_related	Sb04g031590	–	down
bZIP	Sb03g040530	–	down	MYB_related	Sb03g011280	–	down
bZIP	Sb02g027410	–	down	NAC	Sb04g036340	–	up
C2H2	Sb04g021440	–	up	NAC	Sb03g035820	up	–
C2H2	Sb01g031900	up	up	NAC	Sb02g028870	up	down
C2H2	Sb01g031920	up	up	NAC	Sb04g023990	–	down
C2H2	Sb03g025790	–	down	NAC	Sb01g048130	–	down
C2H2	Sb07g028010	–	down	NAC	Sb01g006410	–	down
C2H2	Sb02g028220	–	down	NAC	Sb07g005610	–	down
C2H2	Sb01g043960	–	down	NAC	Sb06g017190	–	down
C2H2	Sb10g004570	–	down	NAC	Sb08g022560	–	down
C3H	Sb03g003110	down	down	NAC	Sb02g043210	down	down
C3H	Sb09g006050	down	–	NF-YB	Sb02g038870	down	down
CO-like	Sb04g029480	–	down	NF-YC	Sb06g033380	up	–
CO-like	Sb06g021480	down	down	Nin-like	Sb04g002940	–	down
EIL	Sb02g043350	–	down	RAV	Sb03g031860	–	down
EIL	Sb04g023730	down	down	TALE	Sb04g008030	–	down
ERF	Sb01g040280	–	up	TALE	Sb05g003750	–	down
ERF	Sb06g025890	up	up	TALE	Sb02g002200	–	down
ERF	Sb06g025900	up	up	TCP	Sb06g023130	up	–
ERF	Sb06g024540	up	up	TCP	Sb10g008030	down	down
ERF	Sb07g023803	up	–	TCP	Sb02g030260	–	down
ERF	Sb03g012890	up	down	Trihelix	Sb03g013050	up	–
ERF	Sb10g004580	down	down	WRKY	Sb03g038170	–	up
ERF	Sb03g042060	–	down	WRKY	Sb10g025600	up	up
ERF	Sb09g020690	–	down	WRKY	Sb10g025590	up	up
ERF	Sb01g044410	–	down	WRKY	Sb09g029850	up	–
FAR1	Sb01g040730	–	down	WRKY	Sb05g001170	up	–
G2-like	Sb07g021290	up	–	WRKY	Sb03g028530	up	down
G2-like	Sb02g001600	up	–	WRKY	Sb03g029920	up	down
G2-like	Sb06g031970	down	down	WRKY	Sb03g047350	down	–
G2-like	Sb01g036680	–	down	WRKY	Sb02g043030	down	down
G2-like	Sb04g003140	–	down	WRKY	Sb04g009800	–	down
GeBP	Sb03g009480	down	down	WRKY	Sb08g005080	–	down
GeBP	Sb03g005180	down	down	WRKY	Sb02g024760	–	down
GRAS	Sb02g034550	–	down	WRKY	Sb01g036180	–	down
GRAS	Sb06g017860	–	down	ZF-HD	Sb05g001690	up	down
GRF	Sb04g034800	–	up				

Interestingly, among the TFs, two *WRKY (Sb03g028530 and Sb03g029920),* one *NAC (Sb02g028870),* one *ERF (Sb03g012890)* and one *ZF-HD (Sb05g00169)* were downregulated in accession 12848 but were upregulated in accession 13443 by low-P stress, while the opposite was observed for one *MYB* (*Sb08g018840*).

### Root development of sorghum in response to low P with and without malate

**Fig. 7:**
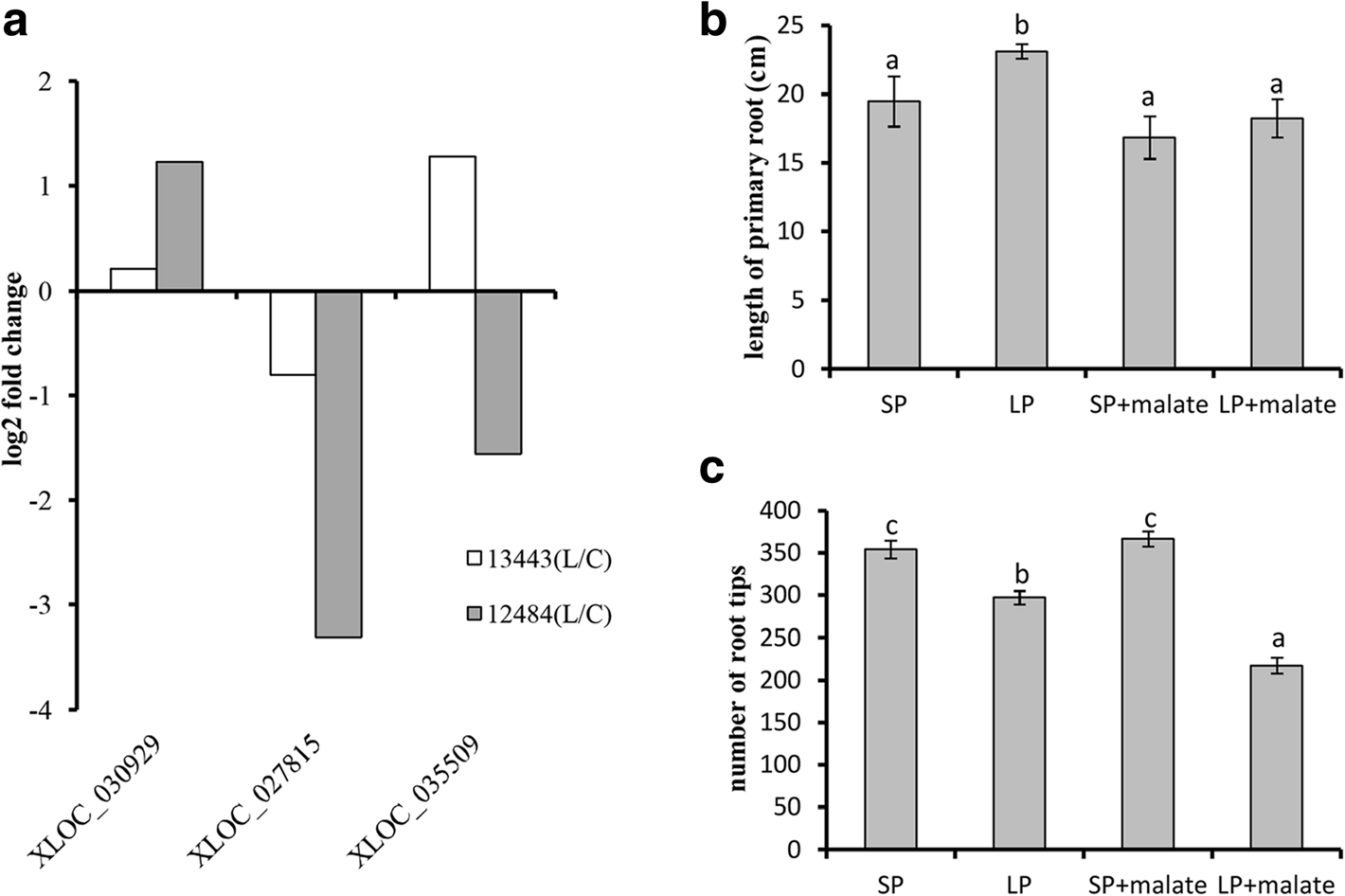
Effect of malate application on root development. **a** Expression profile of the malate metabolism-related genes XLOC_030929 (*Sb07g023910*), XLOC_027815 (*Sb06g020720*) and XLOC_035509 (*Sb09g005810*). **b** Effect of applying malate on the length of the primary root. **c** Effect of applying malate on the number of root tips. The different lowercase letters denote significant differences among different treatments. Bars represent the standard error of the mean (n = 3)

Under low-P stress, plants often secrete organic acid in roots. Among the candidate genes, three were involved in malate metabolism, encoding malate dehydrogenase (Sb07g023910), malate synthase (Sb06g020720) and malic enzyme (Sb09g005810) respectively. And all of them showed different expression patterns in low-P-tolerant and -sensitive accessions in response to low-P stress (Fig. 7a). Our results showed that the primary root length and the numbers of root tips were significantly correlative with P starvation tolerance in sorghum. Therefore, we wondered whether malate affects the primary root length and the number of root tips in sorghum. The results showed that under sufficient-P conditions, application of malate did not affect the length of the primary root and the number of tips significantly (Fig. 7b and c). Under low-P conditions, however, it significantly reduced the length of the primary root and the number of root tips (Fig. 7b and c).

## Discussion

Different sorghum accessions displayed dramatic variability in their tolerance of P starvation, which is associated with shoot internal P content, indicating that P uptake contributes to P starvation tolerance variability. Root morphology is a key trait for optimizing the efficiency of P acquisition in plants [50, 51]. Our results showed that P starvation tolerance in sorghum was significantly correlated with root morphology, implying roots played important roles in P starvation tolerance in sorghum. Therefore, the transcription profiles in sorghum roots were studied. In our study, the majority of the accessions displayed consistent results between experiments carried out in 2 years. However, several accessions, such as 357 and 2349, showed significant differences between the 2 years. This led to the ranking of the tolerance of different accessions to low P. We chose two accessions, 12484 and 13443, as low-P-tolerant and -sensitive varieties, respectively, because these two accessions were constantly at the two extremes of the tolerance ranking. The ranking variations of tolerance to low P were concurrent with those of internal P content. The variations in internal P content of the same accession in different years indicate that there are other environmental factors beyond genotype contributing to P acquisition.

### The two accessions showed great differences in root morphology under low-P stress

It was suggested that most plant responses to P deficiency included the remodeling of root morphology, which involves suppressing primary root growth and enhancing the production of root hairs and lateral roots [52–54]. In our study, both of the selected materials showed enhanced primary root elongation under low-P stress (Fig. 3), while more lateral roots were promoted in tolerant accessions than sensitive accessions, implying that the genes regulating the elongation of the primary root and the enhancement of lateral roots play important roles in the response to low-P stress. It is well documented that root morphology is regulated by many signaling pathways. For instance, the AUX signal-related genes *OsARF12* and *OsTOP1* were involved in primary root development in rice [55, 56]. The SA signal-related gene *OsAIM1* and GA signal-related gene *OsSHB* were also involved in primary root development [57, 58]*,* while the MYB type TF *OsMYB1* was involved in lateral root development [59]. Root hairs play key roles in P uptake in plants [60]. In Arabidopsis, some TFs, including MYB types such as MEMBRANE ANCHORED MYB (maMYB) [61], bHLH types such as RHD SIX-LIKE 1 (RSL1) [62], RHD SIX-LIKE 3 (RSL3) [63], *Lotus japonicus* ROOT HAIR LESS-LIKE 2 (LRL2) [64] and HD-ZIP types such as HOMEODOMAIN GLABROUS 11 (HDG11) [65] were found to be involved in root hair growth. Moreover, P starvation-induced genes involved in root hair development have been identified. For instance, *OsAUX1* encoding an auxin influx transporter could promote root hair elongation under low-P stress in rice [66]. In Arabidopsis, the ET signaling gene EIN3 and its closest homolog EIL1 were demonstrated to be involved in P starvation-induced root hair development [67]. In our study, we found two EIL proteins, Sb02g043350 and Sb04g023730, and three bHLH-type TFs, Sb03g008290, Sb01g043570 and Sb10g005650, similar to RSL1, RSL3 and LRL2, respectively, that were candidate genes, suggesting that these genes might regulate P tolerance by mediating root hair growth in sorghum. Thus, our data help reveal the related genes or mechanism controlling root morphology in sorghum under low-P stress.

### More active responses to P starvation in the low-P-tolerant accession

Under different P treatments, the number of DEGs in the low-P-tolerant accession 12484 was nearly two-fold higher than that in the low-P-sensitive accession 13443, implying that the low-P-tolerant accession responded to low P more positively and dramatically. Moreover, the numbers of unique DEGs identified in accession 13443 or accession 12484 were greater than the number of overlapping DEGs, indicating that there are differences between the two accessions in response to low P. Similarly, many more DEGs in response to low P were found in low-P-tolerant accessions than in low-P-sensitive accessions in soybean [68]. In maize, however, the number of DEGs in low-P-tolerant materials was lower than that in low-P-sensitive materials under low-P stress [69]. Thus, it is not valid to evaluate whether an accession is tolerant of low P or not solely based on the number of DEGs under low-P conditions.

### Complex mechanism of P starvation tolerance in sorghum

Among the candidate genes, the significantly enriched GO terms referred to molecular function, cellular component and biological process, implying that achieving P starvation tolerance required many processes. The top 30 significantly enriched GO terms of DEGs under low-P conditions suggested that the two accessions had differences in molecular functions under low-P conditions. Further analysis showed that the input frequency of antioxidant activity, catalytic activity, transporter activity and nucleic acid binding TF activity, belonging to molecular function, were higher than the background frequency, suggesting the key roles of genes belonging to these terms in low-P tolerance in sorghum.

Candidate genes related to P starvation tolerance were enriched in many pathways. Notably, genes participating in phenylpropanoid biosynthesis, secondary metabolism, starch and sucrose metabolism and nitrogen metabolism also actively responded to low-P stress in maize [39], Arabidopsis [70] and rice [71]. Phenylpropanoid biosynthesis, phenylalanine metabolism, and biosynthesis of secondary metabolites were the top 3 enriched pathways, suggesting these pathways responded actively when sorghum suffered from low-P stress. Phenylpropanoids contribute to all aspects of plant responses toward biotic and abiotic stimuli, and phenylpropanoids are not only indicators of plant stress responses under varied light, mineral treatment, and pest stresses but are also key mediators in plants [72, 73]. The same results were also reported in soybean under low-P stress [53]. Moreover, the candidate genes were significantly enriched in flavone and flavonol biosynthesis, carotenoid biosynthesis and flavonoid biosynthesis pathways. Flavonol is one of the main classes of phenylpropanoid pathway derivatives [74–76]. Flavonoids and carotenoids are widely known for their influence on the colors of plant tissues, which further contributes to plant fitness and food quality. The flavonoid pathway has been suggested to play important roles in protecting plants from oxidative stresses induced by drought [77], temperature [78], and nitrogen, P, or carbon nutrition [79, 80]. Carotenoids are crucial for driving biological processes in plants, including the assembly of photosystems and light harvesting antenna complexes for photosynthesis and the regulation of growth and development [81, 82]. A kind of carotenoid derivative, strigolactone, plays roles in root responses to low-P stress in Arabidopsis [83] and tomato [84].

In general, the results of our study show that many GO terms and pathways are enriched in sorghum suffering from low-P stress, implying that a complex mechanism of P starvation tolerance exists in sorghum.

### Malate play key roles in root development in sorghum under low-P stress

Notably, we found that applying malate reduced the length of the primary root and the number of root tips under P stress, implying malate was involved in P starvation tolerance in sorghum by affecting root development. Recently, reactive oxygen species (ROS) were also found to trigger callose deposition, which further adjusts apical meristem activity in roots under low-P stress [85, 86]. For the candidate DEGs, many GO terms related to oxidoreductase activity were found, which suggested that ROS generation and elimination were active in sorghum suffering from low-P stress. In our study, applying malate only affected root development under low-P conditions, suggesting that low-P stress might generate some factors that perhaps promote malate to affect root development. Moreover, in Arabidopsis, malate was found to inhibit root growth by adjusting the accumulation of Fe in plants under low-P stress, which is dependent on ALMT [43]. It has also been reported that Fe overloading modifies root growth in P-deprived plants [87–89]. Additionally, ROS is produced during low-P stress with the accumulation of Fe^3+^ [85, 90]. Our GO enrichment results showed that many P candidate genes were involved in Fe binding. From these results, we speculate that malate plays key roles in root development in sorghum under low-P stress, perhaps through Fe and ROS.

### Plant hormone signal transduction and TFs related to P starvation tolerance in sorghum

Plant hormones are involved in many biological processes in enhanced resistance to environmental stresses, diseases and pathogen infections [91]. In the present study, many DEGs were related to plant hormone (including AUX, ABA, JA, ET, and SA) signal transduction. AUX has been suggested to stimulate root growth and lateral root proliferation upon P starvation [7]. Notably, the 3 *AUX/IAA* genes (*Sb03g001490, Sb03g035500, Sb06g023800*) related to AUX signal transduction were downregulated in low-P-tolerant material by low-P stress but were not significantly changed in low-P-sensitive material. In Arabidopsis, the phloem-mobile transcript of IAA could target the root tip and then regulate root morphology [92]. We speculate that the 3 *AUX/IAA* genes play similar roles in root morphology regulation, but the specific functions of these genes under low-P stress in sorghum should be further studied.

ET was also involved in low-P stress. P starvation could alter ET biosynthesis in plants, while ET could regulate root growth under low-P stress [93–95]. In our study, several ET signal-related genes were considered to be related to low-P tolerance in sorghum. In Arabidopsis, the *eto1* mutant overproducing ET exhibited reduced primary root growth and increased production of root hairs [96]. Whether a similar regulatory mechanism also exists in sorghum should be further investigated.

Although the exact roles of ABA and SA in plants suffering from low P are not clear, these hormones were found to respond to low P. In Arabidopsis, ABA biosynthesis mutants exposed to low-P stress displayed reduced expression of P starvation-responsive genes and accumulation of anthocyanins [97]. Some studies have suggested that SA is involved in the response of plants to low P by controlling their redox status [98, 99]. In the present study, two candidate *bZIP* genes (*Sb03g040530, Sb09g021840*) encoding TGA were involved in the SA signaling pathway. In Arabidopsis, type-II TGA TFs were demonstrated to be key activators of JA/ET-induced immune reactions [100]. JA accumulation occurs when plants are exposed to a low-P environment, further enhancing herbivory resistance and reducing shoot growth [101]. However, the role that SA plays in low-P stress is unclear. Thus, we speculate that SA can regulate P starvation through crosstalk with the JA/ET signaling pathway or other pathways, but the underlying mechanism should be further studied.

TFs were suggested to play a key role in the regulation of gene expression at the transcriptional level by binding to DNA regulatory elements [102]. Substantial evidences have proved that TFs are also involved in phosphate homeostasis [103–106]. In our study, besides ET- and SA-related TFs, many other TFs including bHLHs, WRKYs, MYBs, bZIPs, NACs and C2H2 zinc finger proteins, were identified. In rice, *OsMYB2P-1* is involved in the regulation of P starvation responses and root architecture by suppressing or activating downstream genes [107]. Overexpression of OsMYB4P could activate the expression of several Pht genes and increase phosphate acquisition [108]. WRKY74 modulates the tolerance to phosphate starvation in rice [109]. In wheat, *TaZAT8*, a C2H2-ZFP-type TF gene, plays critical roles in mediating wheat tolerance to P deprivation by regulating P acquisition, ROS homeostasis and root system establishment [110]. Our results indicate that TFs may also play important roles in sorghum tolerance to low P, but their functions should be further studied.

## Conclusions

Transcriptome analysis showed that a P starvation-tolerant accession exhibited more active responses to low-P stress. A total of 2089 genes were identified and enriched in many GO terms and pathways, suggesting that P starvation tolerance of sorghum is a complex mechanism. Malate significantly reduced the length of the primary root and numbers of root tips in sorghum suffering from low-P stress. Plant hormone (including AUX, ET, JA, SA and ABA) signal transduction-related genes and many TFs were found to be involved in low-P tolerance in sorghum. The findings reported herein increase our understanding of the molecular characteristics of sorghum tolerant of low-P stress. The identified accessions will be useful for inbreeding new sorghum varieties with a high P starvation tolerance.

## Additional files

Additional file 1: **Table S1.** Gene-specific primers used in gene expression analysis by qRT-PCR. (XLSX 13 kb)

Additional file 2: **Figure S1.** GO annotations and GO enrichment of DEGs. a and e DEGs in accession 12484 in response to low-P stress. b and f DEGs in accession 13443 in response to low-P stress. c and g DEGs in different accessions under low-P conditions. d and h Active DEGs. DEGs in accession 12484 in response to low-P stress. (PDF 1359 kb)

## Data Availability

The datasets supporting the conclusions of this article are included within the article and its additional files.

## Abbreviations

ABA: Abscisic acid
AUX: Auxin
*COI1*: *Coronatine insensitive 1*
DEG: Differentially expressed gene
ET: Ethylene
FDR: False discovery rate
FPKM: Fragments per kilobase of transcript per million fragments mapped
GO: Gene ontology
JA: Jasmonic acid
*JAZ*: *Jasmonate ZIM-domain*
KEGG: Kyoto encyclopedia of genes and genomes
MYB62: MYB domain protein 62
P: Phosphorus
PHR1: Phosphate starvation response1
PHT: Phosphate transporter
Pi: Inorganic orthophosphate
*PP2C*: *Protein phosphatase 2C*
*PSTOL1*: *Phosphorus starvation tolerance 1*
qRT-PCR: Quantitative real-time polymerase chain reaction
R/S ratio: Root to shoot weight ratio
RADR: Relative average diameter of root
RDWR: Relative dry weight of root
RDWS: Relative dry weight of shoot
RLR: Relative length of roots
RNA-Seq: mRNA sequencing
RNRT: Relative number of root tips
ROS: Reactive oxygen species
RPCS: Relative P content of shoot
RSAR: Relative surface area of root
RVR: Relative volume of root
SA: Salicylic acid
*SAUR*: *Small auxin upregulated RNA*
TF: Transcriptional factor
