# Supplementary figures and images for: Transcriptome analysis reveals candidate genes related to phosphorus starvation tolerance in sorghum
Source: BMC Plant Biol. 2019 Jul 11;19:306. doi: 10.1186/s12870-019-1914-8 (PMC6624980; doi:10.1186/s12870-019-1914-8)

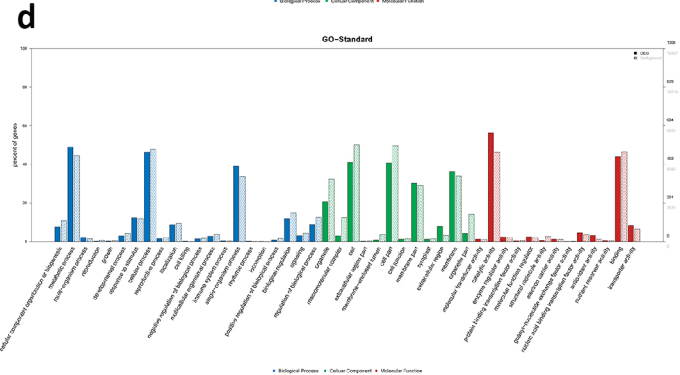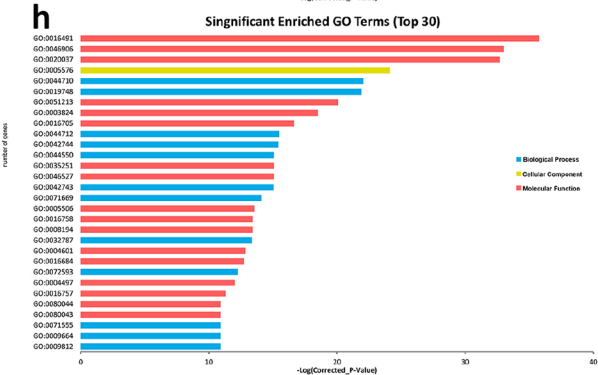

Supplement: Supplementary file 2 — Figure S1. GO annotations and GO enrichment of DEGs. a and e DEGs in accession 12484 in response to low-P stress. b and f DEGs in accession 13443 in response to low-P stress. c and g DEGs in different accessions under low-P conditions. d and h Active DEGs. DEGs in accession 12484 in response to low-P stress. (PDF 1359 kb) [file 12870_2019_1914_MOESM2_ESM.pdf]
